# D-Pinitol improves cognitive dysfunction and neuronal damage induced by isoproterenol via modulation of NF-κB/BDNF/GFAP signaling in Swiss albino mice

**DOI:** 10.22038/IJBMS.2023.72207.15698

**Published:** 2024

**Authors:** Aamir Khan, Sumit Sharma, Anwesha Das, Mumtaz Alam, Mansoor Ali Syed, Syed Ehtaishamul Haque

**Affiliations:** 1 Department of Pharmacology, School of Pharmaceutical Education and Research (SPER), Jamia Hamdard, New Delhi–110062, India; 2 Drug Design and Medicinal Chemistry Lab., Department of Pharmaceutical Chemistry, School of Pharmaceutical Education and Research (SPER), Jamia Hamdard, New Delhi–110062, India; 3 Department of Biotechnology, Jamia Millia Islamia, New Delhi 110025, India

**Keywords:** D-Pinitol, Inflammation, Isoproterenol, Neuroprotection, Neurotoxicity, Oxidative stress

## Abstract

**Objective(s):**

Neurological disorders are the world’s most distressing problem. The adverse effects of current medications continue to compel scientists to seek safer, more effective, and economically affordable alternatives. In this vein, we explored the effect of D-Pinitol on isoproterenol-induced neurotoxicity in mice.

**Materials and Methods:**

Forty-two mice were randomly distributed into 7 groups each having 6 animals. Group I; received saline. Group II; received isoproterenol (ISO) 15 mg/kg/day, s.c. for 20 days. Group III, IV; received 50 and 100 mg/kg/day/oral of D-Pinitol, respectively along with ISO for 20 days. Group V; received D-Pinitol 100 mg/kg/day/oral for 20 days. Group VI; received propranolol 20 mg/kg/day/oral and ISO for 20 days. Group VII; received propranolol 20 mg/kg/day/oral for 20 days. On the 21st day after behavioral tests, blood was collected and mice were sacrificed for various biochemical, histopathological, and immunohistochemical analyses.

**Results:**

Chronic administration of isoproterenol caused neurotoxicity, cognitive dysfunction, and histopathological changes in the brain as evidenced by increase in GFAP, oxidative stress (via SOD, CAT, TBARS, and GSH), neuroinflammation (NF-kB, TNF-α, IL-6, and IL-10), and decrease in AchE and BDNF. Co-administration of D-Pinitol (100 mg/kg) significantly prevented these pathological alterations. The cognitive improvement was also observed through the forced swim test, elevated plus maze test, and rotarod test.

**Conclusion:**

Our findings on D-Pinitol thus clearly established its neuroprotective role in ISO-induced neurodegeneration in Swiss albino mice.

## Introduction

Evidence suggests that neuroinflammation and oxidative stress are responsible for implications in the pathogenesis of neural diseases like Alzheimer’s disease, Parkinson’s disease, depression, and memory loss (1). Isoproterenol (ISO), a β adrenergic receptor agonist, is used in asthma, bradycardia, mild heart block, and bronchitis (2). Previous studies reported that ISO mediates the generation of free radicals through activation of NADPH oxidase which causes oxidative stress and inflammatory reaction in the brain. Elevation in the production of reactive oxygen species (ROS) stimulates the nuclear factor kappa-B (NF-κB). ROS elevate the generation of inflammatory and pro-inflammatory cytokines in glial cells. Up-regulation of NF-κB and elevation of cytokines are involved in the pathway of cellular inflammation (3, 4). Brain-derived neurotrophic factor (BDNF) is a type of neurotrophin that plays a key part in cognitive function and also in the pathology of Alzheimer’s disease and Huntington’s disease (5). It has been reported that chronic activation of NF-kB, which causes neuroinflammation, results in a decrease in BDNF levels, which correlates with cognitive dysfunction and neurodegeneration (6). Thus, ROS are responsible for initiating cerebral injury. ISO inhibits the activity of acetylcholinesterase (AchE), a brain-specific enzyme, responsible for the catalysis of acetylcholine into acetate and choline. Alteration of neurotransmitter concentration in the brain can cause behavioral changes, cognitive deficiency, and neurodegeneration (7, 8). Glial fibrillary acidic protein (GFAP) is a monomer filament protein that is found in glial cells. GFAP is a specific biological marker for the prediction of brain injury (9). It is reported that neuronal injury due to oxidative stress triggers the activation of glial cells and up-regulates GFAP (10).

Natural products are valuable reservoirs of bioactive nutrients and have important contributions to pharmacotherapy for various diseases including neurological disease. Furthermore, previous studies reported that the anti-oxidant and anti-inflammatory properties of bioactive nutrients ameliorate cognitive function and degenerative processes in the brain (11, 12). D-Pinitol (PIN), a cyclitol, is a bioactive compound having molecular formula C_7_H_14_O_6 _and is present in many plant species. PIN is chiefly found in soybean (*Glycine max *L Merr.) and carob pod (*Ceratonia siliqua* L). It has been previously reported that PIN has different pharmacological properties like anti-diabetic, anti-oxidant, anti-inflammatory, chemopreventive, anti-hepatotoxic, and anti-osteoporosis effects (13 - 15). Based on these therapeutic benefits, we planned our study to investigate the potential prophylactic effect of PIN against ISO-induced neurotoxicity and behavioral changes in Swiss albino mice. Additionally, the possible mechanisms of PIN in brain tissues, including anti-oxidant and anti-inflammatory effects were also studied.

## Materials and Methods

**Experimental animals**

We obtained 42 mice around 30–50 g from our institution through IAEC-approved project number 1837 on 10/02/202. All mice were acclimatized for 7 days prior to the investigation at 23.0 °C ± 2.0 °C and around 60 percent ± 5 percent humidity. All animals have unrestricted access to standard pallet diet and water. The experiments were performed as per CPCSEA guidelines.

**Drugs and chemicals**

D-Pinitol and propranolol with chemical abstracts service No.10284-63-6 and 318-98-9, respectively from Tokyo chemical industry (India) and Isoproterenol No 5984-95-2 from Merck (Sigma-Aldrich Solutions), India were procured. For BDNF (Brain-derived neurotrophic factor), TNF-α, IL-6, and IL-10, ELISA kits were procured from Krishgen Biosystems, India. Water and chemicals of analytical grade were employed for various biochemical assessments.

**Experimental protocol**

Forty-two male mice were randomly distributed into 7 groups each having 6 animals. Group I (control); received 0.5 ml/kg saline, orally for 20 days. Group II (toxic control); received 15 mg/kg/day isoproterenol (ISO) subcutaneously (SC) for 20 days. Group III-IV (PIN-treated group); received 50 and 100 mg/kg/day/oral of PIN, respectively along with ISO (15 mg/kg/day, SC) for 20 days. Group V (PIN *per se* group); received PIN (100 mg/kg/day/oral) for 20 days. Group VI (PRO treated group); received propranolol 20 mg/kg/day/oral along with ISO (15 mg/kg/day, SC) for 20 days. Group VII (PRO *per se *group); received propranolol (20 mg/kg/day/oral) for 20 days. On the 21^st^ day behavioral tests were performed. Following the behavioral tests, mice were anesthetized and blood was taken for various estimations. The mice were then sacrificed by CO_2_ inhalation, and their hippocampus and cortex were taken for biochemical, histological, and immunohistochemical analysis. 

**In silico investigation**

Auto Dock 4.0 software (version 1.5.6) was used for molecular docking. 3D conformer of D-Pinitol was obtained from Pub Chem in .sdf format, and Chem Bio 3D Ultra 14.0 was used for energy minimization. The GFAP protein crystal structure (PDB ID: 6A9P, resolution: 2.51, https://www.rcsb.org/structure/6A9P) was derived from the RCSB PDB database. Before starting the docking procedure, the B, C, D, E, F, G, and H chains of the protein were deleted as they were part of the GFAP homo octamer. Using Auto Dock Tools, the protein was engineered by eliminating heteroatoms and co-crystallized water molecules, adding missing atoms, polar hydrogens, and Kollman charges. “Blind docking” methodology was considered, i.e., chain A was considered for molecular docking. The grid box was created utilizing 126*126*126 grid points, with the grid center set to -1.117, -283.339, and 816.820 for X, Y, and Z coordinates, respectively, at 1.000 Å spacing to engulf both chains. The Lamarckian Genetic Algorithm (LGA) was selected for the best conformer searches, and a maximum of 25 conformers were examined. The protein-compound binding interactions were visualized using BIOVIA Discovery Studio Visualizer 2019, version 19.1.0.18287.

**Evaluation of behavioral parameters**

***Forced swim test (FST)***

An open cylindrical container with 45 cm × 15 cm × 45 cm dimension was used for this test. The container was filled with water (temperature 24 ± 5 °C) approximately 35 cm in height. Each mouse was forced to swim for 15 min pretest. After 24 hr, each mouse was re-exposed to the same swimming environment as described above for 5 min of FST. Fresh water was used for each trial. Total swimming time, climbing time, and immobility time were recorded. Swimming was described as the horizontal motion of the animal within the container and immobility was no additional movement or making slight movement (16).

***Elevated plus-maze (EPM) test***

An EPM tool (with open and closed arms) was used to carry out this test. This apparatus was kept 30 cm above the ground in a quiet place with proper light. For this test, each mouse was kept at the center of the plus maze apparatus facing towards the open arms. The time spent in each arm and the frequency of entries were recorded (17).

***Rotarod test***

The Rotarod test was performed for the assessment of learning and motor coordination. Before testing, each mouse went through the training session. For training, each mouse was placed on the rotating bar of the rotarod at the speed of 4 rpm for 60 sec. This procedure was repeated three times separated by 10 min intervals. After training, the rotarod test was performed by putting each mouse on the rotarod bar at 8 rotations per min up to 120 sec. The time of persistence on the rotating bar was recorded for each mouse (18).

**Evaluation of biochemical parameters**

***Preparation of tissue homogenate***

After sacrificing the animals, the brain was collected and washed using cold saline. The hippocampus part was segregated and homogenized using cold 0.1 M phosphate buffer with pH 7.4. The homogenate was then centrifuged at 10,000 g for 10 min at 4 °C, and the supernatant was utilized for different biochemical parameters.

***Evaluation of oxidative stress markers***

*Estimation of catalase activity (CAT)*

The activity of Catalase was estimated using the method given by Claiborne, 1986 (19). The supernatant was added to an H_2_O_2 _solution made in potassium phosphate buffer, and absorbance was read for 3 min at 240 nm at an interval of one minute each.

*Estimation of superoxide dismutase (SOD) activity*

The SOD activity was estimated according to the standard method given by Marklund (20). The supernatant of the hippocampus homogenate was gently mixed with Tris-HCL buffer with pH 8.5. To this mixture pyrogallol was mixed and absorbance was observed at 420 nm wavelength.

*Estimation of reduced glutathione (GSH)*

A previously published approach was used to evaluate reduced glutathione activity (21). The supernatant (2 ml) from the homogenate was mixed with a mixture of Tris buffer (4 ml) and DTNB (0.1 ml). Absorbance was read at 410 nm.

*Evaluation of lipid peroxidation*

Lipid peroxidation was estimated using a previously published method (22). One milliliter of 10% tissue homogenate was mixed with a reagent (0.5 ml TCA + 0.5 ml TBA). This mixture was centrifuged and the supernatant was collected. Absorbance was observed at 540 nm wavelength against the blank.

*Estimation of inflammatory markers*

Inflammatory markers (TNF-α, IL-6, and IL-10) were evaluated using commercial ELISA kits in accordance with the manufacturer’s instructions. ELISA kit for TNF-α was purchased from Elabscience Biotechnology Co., Ltd, USA (TNF-α, cat # E-EL-M3063) and ELISA kits for IL-6 and IL-10 were procured from Krishgen, Worli, India (IL-6, cat # KB2068; IL-10 cat # KB2072). The inflammatory cytokines in tissue homogenate were estimated using a standard curve and represented in pg/ml.

*Estimation of acetylcholinesterase (AchE) and BDNF*

The level of Acetylcholinesterase (AchE) was calculated using Elman’s assay method (23). 0.2 ml of supernatant was added with 75 mM Acetylthiocholine Iodide (0.2 ml), buffered DTNB (0.1 ml) (Elman’s reagent), and 3 ml of 25 mM Phosphate buffer (pH 7.4). Then incubated for 10 min at 77 °F. The absorbance was observed at 412 nm. The level of BDNF was estimated using an ELISA kit as per the manufacturer’s instructions. The BDNF ELISA kit was obtained from Krishgen, Mumbai, India.

**Histopathological analysis (H & E staining)**

The mice’s brains were extracted after euthanasia, washed in normal saline, and dried. 10% formalin solution was used to keep the brain tissue for H & E staining. After staining the tissue with Haematoxylin and Eosin, paraffin slices of brain tissues were prepared (24). Photomicrographs of brain sections were taken using a Motic microscope.

**Immunohistochemistry (IHC) analysis of NF–κB p65 and GFAP**

Immunohistochemical evaluation was done to enumerate the expressions of NF–κB p65 and GFAP protein in the brain tissue. From the prepared paraffin blocks of brain tissues, 10 μm thin sections were transversely cut and paraffin was removed by xylene, then dehydrated with ethanol, and proceeded as per the established method (25).

***Statistical analysis***

One-way ANOVA for statistical analysis and Tukey’s test for determination of significance among different groups were used. Values were expressed as mean ± SEM (standard error mean) for n=6. Graph Pad Prism software (version 8.0.1) was used for statistical analysis.

## Results

***Evaluation of in silico analysis***

**Figure 1:**
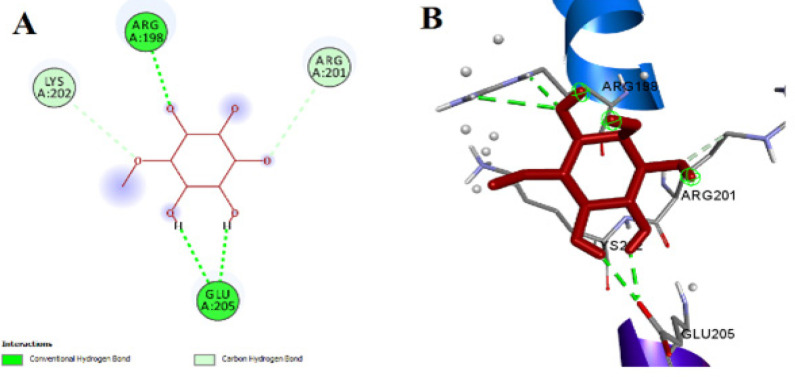
Representative images showing

We performed molecular docking of the test drug (D-Pinitol) with GFAP. PIN binds with the active sites of GFAP with -1.26 Kcal/mol binding energies. PIN also expected promising orientations within the binding site of GFAP. PIN’s docked pose detected the conventional hydrogen bond interactions with amino acid Arg198, Glu205 and interactions of carbon hydrogen bond with amino acid Lys202, Arg201 at the GFAP binding site. Thus PIN indicated the binding affinity for GFAP. The docking image of the D-Pinitol-GFAP complex is shown in Figure 1.

***Effect of D-Pinitol on FST***

**Figure 2:**
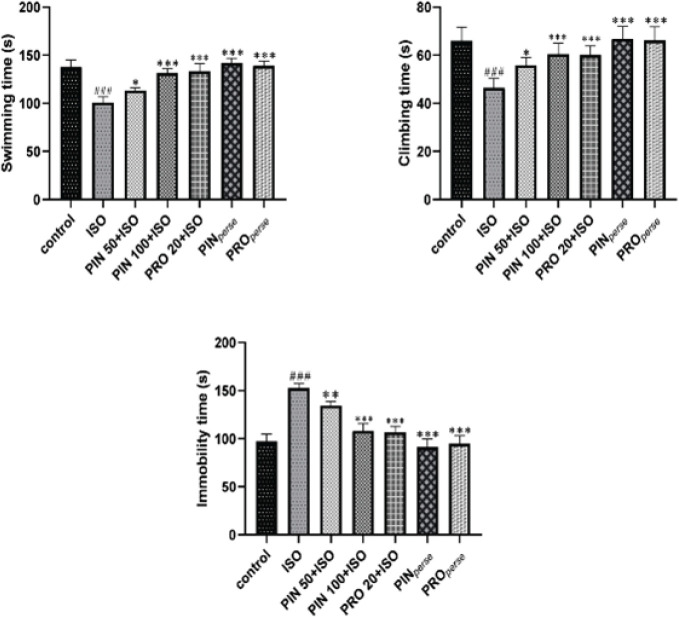
Effect of D-pinitol on FST in ISO-induced neurological toxicity in mice

The toxic control (ISO) group represents the notable escalation in the immobility time (*P*<0.001) and decreased swimming and climbing time (*P*<0.001) in comparison to the control group. Treatment with 100 mg/kg of PIN and 20 mg /kg of propranolol significantly decreased the immobility time (*P*<0.001, each) and elevated the swimming and climbing time (*P*<0.001, respectively) in comparison to treatment with ISO. The low dose of PIN (PIN 50) produced a less significant effect as compared to the PIN 100. *Per se* groups indicated almost the same outcome as that of the control group (Figure 2).

***Effect of D-Pinitol on EPM***

**Figure 3:**
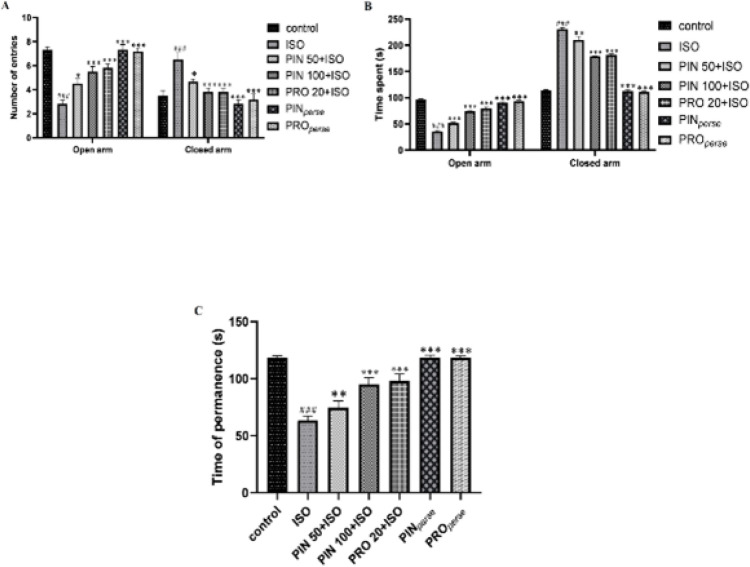
Effect of D-pinitol on EPM and rotarod test in ISO-induced neurotoxicity

ISO-treated mice indicated a reduction in the number of entries and time spent (*P*<0.001) in the open arm and a notable escalation in number of entries and time spent (*P*<0.001) in the closed arm in comparison to the control group. PIN 50 and PIN 100 treated groups showed significant increase in number of entries (*P*<0.05, *P*<0.001, respectively) and time spent (*P*<0.001, each) in the open arm and notable reduction in number of entries (*P*<0.05, *P*<0.001, respectively) and time spent (*P*<0.01, *P*<0.001, respectively) in the closed arm in comparison to ISO group. PRO 20 group also produced a notable increment in number of entries (*P*<0.001) and time spent (*P*<0.001) in the open arm and notable reduction in the number of entries (*P*<0.001) and time spent (*P*<0.001) in the closed arm in comparison to ISO treated group (Figures 3A and 3B).

***Effect of D-Pinitol on rotarod test***

In comparison to the control group, treatment with ISO represents a notable reduction in time on permanence (*P*<0.001) on the rotarod at 8 rpm. 50 mg/kg and 100 mg/kg dose of PIN treated group notably increased the time on permanence (*P*<0.01, *P*<0.001, respectively) as compared to the ISO group. 20 mg/kg propranolol-treated group also showed a notable increment in time on permanence (*P*<0.001) as compared to the ISO-treated group (Figure 3C).

***Effect of D-Pinitol on hippocampus oxidative stress markers***

**Figure 4:**
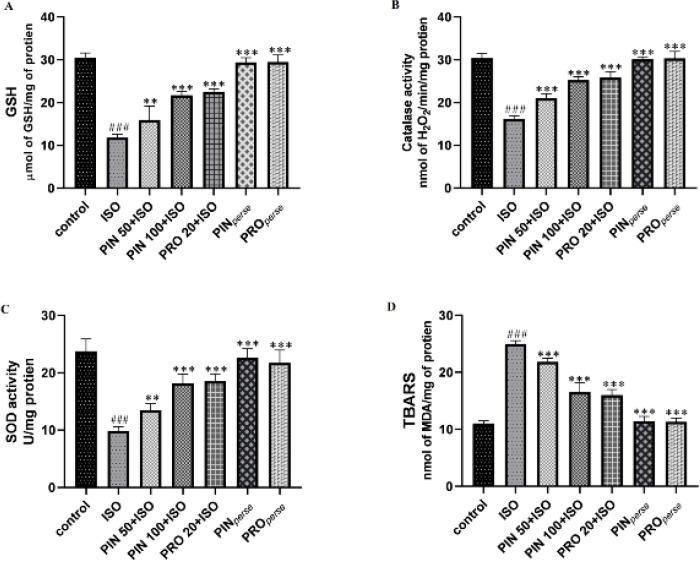
D-pinitol showing the protective effect on oxidative stress caused by ISO

Treatment with ISO indicated a notable reduction in catalase, superoxide dismutase activity, and GSH level, with increase in the MDA level, in comparison to the control group (*P*<0.001). Treatment with 50 mg/kg and 100 mg/kg D-Pinitol showed a significant increase in anti-oxidant enzymes level, i.e., CAT (*P*<0.001, each), SOD (*P*<0.01, *P*<0.001, and *P*<0.001, respectively) and GSH (*P*<0.01, *P*<0.001, and *P*<0.001, respectively) and reduction in TBARS level (*P*<0.001, each), when compared to the ISO group. *Per se *groups produced nearly the same results as the control group (Figure 4).

***Effect of D-Pinitol on hippocampus cytokine levels***

**Figure 5:**
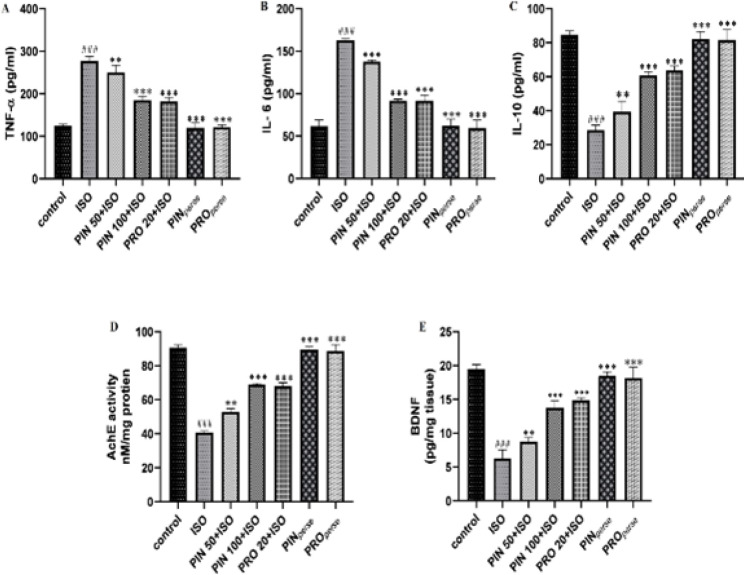
Effect of D-pinitol on inflammatory markers, AchE and BDNF, in Swiss albino mice

Treatment with ISO showed notable elevation in TNF-α and IL-6 and decreased IL-10 (*P*<0.001) in the hippocampus of mice in comparison to the control group. PIN 50 and PIN 100 treated groups notably had reduced levels of TNF-α (*P*<0.01, *P*<0.001, respectively) and IL-6 (*P*<0.001) and increased IL-10 (*P*<0.01, *P*<0.001, respectively) level as compared to the ISO group. PRO 20 also reverted these cytokines towards the normal level (*P*<0.001) (Figure 5).

***Effect of D-Pinitol on hippocampus AchE and BDNF levels***

ISO treatment significantly decreased the AchE and BDNF levels (*P*<0.001) in comparison to the control group. In comparison to the ISO group, PIN 50, PIN 100, and PRO 20 groups showed notable elevation in AchE (*P*<0.01, *P*<0.001, and *P*<0.001, respectively) and BDNF (*P*<0.01, *P*<0.001, and *P*<0.001, respectively) towards the normal. *Per se* groups indicated similar results to the control group (Figure 5).

***Histopathological evaluation by H*** ***&*** ***E staining***

**Figure 6:**
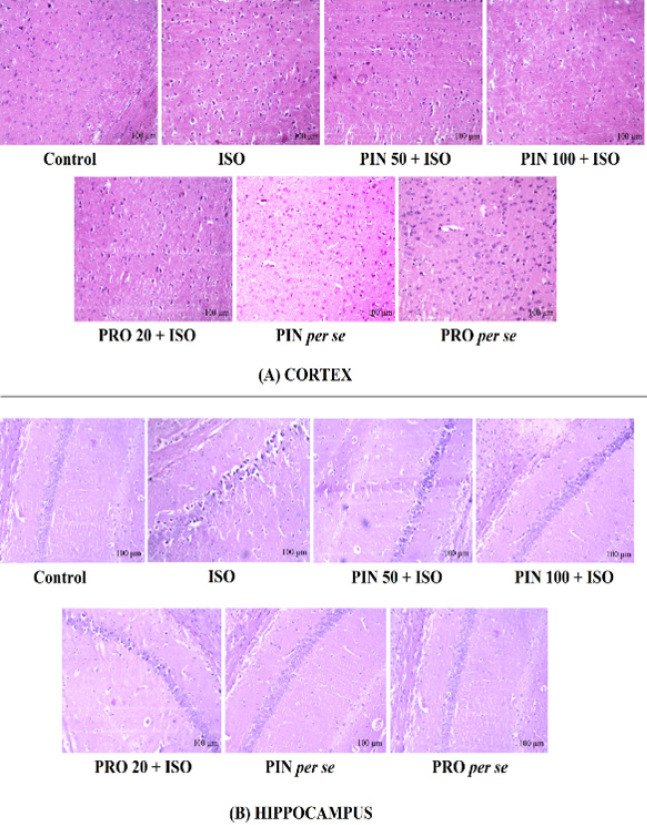
Photographs of (A) cortex and (B) hippocampus showing histological alterations in different groups

As shown in Figure 6, pyknosis, vacuolization, and degeneration of neurons were seen in the hippocampus of ISO treated group. Treatment with PIN 100 and PRO 20 demonstrated a notable decrease of these damages towards the normal, whereas, PIN 50 showed less significant effect as compared to PIN 100 group. Control and *per se *groups demonstrated normal histology of the hippocampus and cortex regions (Figure 6).

.

***Effect of D-Pinitol on the expression of NF–κB p65 and GFAP***

**Figure 7:**
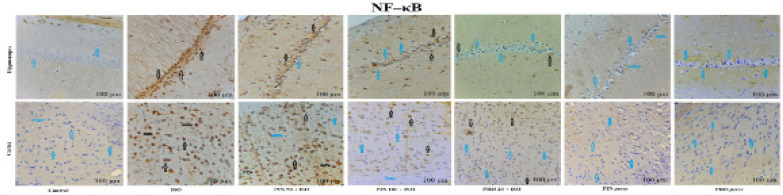
Images displaying the effect of D-pinitol on NF–κB expression in the cortex and hippocampus by immunohistochemical staining

**Figure 8:**
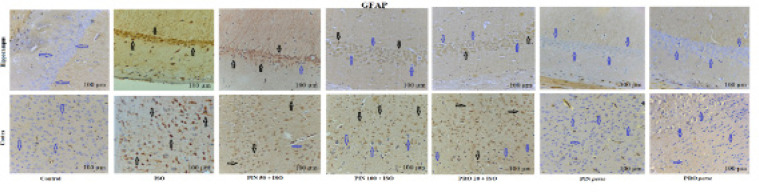
Images displaying the effect of different doses of D-pinitol on the expression of GFAP in the hippocampus and cortex by immunohistochemical staining

The outcome of PIN on the levels of NF–κB p65 and GFAP in the cortex and hippocampus by immunohistochemical staining is shown in Figures 7 and 8, respectively. Results showed that treatment with ISO notably elevated the NF–κB p65 and GFAP expression in cortex and hippocampus tissue in comparison to the control group. PIN (100 mg/kg) and propranolol (20 mg/kg) treated groups displayed a notable decrease in NF–κB p65 and GFAP expression in comparison to the ISO group. Treatment with PIN 50 demonstrated smaller effects in comparison to PIN 100. The *per se* group did not show any notable effects in NF–κB p65 and GFAP expression (Figures 7 and 8).

A. 2D interaction diagram and B. 3D interaction diagram of D-pinitol-GFAP complex (PDB ID: 6A9P).

Data is presented as mean ± SEM (n=6). *P*<0.05 is considered significant. ###P˂0.001 vs control; *P˂0.05, **P<0.01, ***P<0.001 vs ISO.

FST: Forced swim test; ISO: Isoproterenol

Figures A and B show the results of the EPM. Figure C shows the results of the rotarod test

Data is presented as mean ± SEM (n=6). *P*<0.05 is considered significant. ###*P*˂0.001 vs control; *P˂0.05, **P<0.01, ***P<0.001 vs ISO.

EPM: Elevated plus maze; ISO: Isoproterenol

(A) GSH level, (B) CAT activity, (C) SOD activity, and (D) TBARS level.

Data is presented as mean ± SEM (n=6). *P*<0.05 is considered significant. ###*P*˂0.001 vs control; *P˂0.05, **P<0.01, ***P<0.001 vs ISO.

ISO: Isoproterenol

(A) TNF-α, (B) IL-6, (C) IL-10, (D) AchE, and (E) BDNF levels are shown

Data is presented as mean ± SEM (n=6). *P*<0.05 is considered significant. ###*P*˂0.001 vs control; *P˂0.05, **P<0.01, ***P<0.001 vs ISO.

AchE: Acetylcholinesterase; BDNF: Brain-derived neurotrophic factor

ISO group shows degeneration of neurons, vacuole formation, and pyknotic nucleus. D-pinitol and propranolol-treated groups show notable reductions in these histopathological changes. The per se group did not show altered neuronal architecture. [Scale bar 100 µm].

ISO: Isoproterenol

Blue arrows represent the healthier neurons while black arrows represent NF–κB staining [Scale bar 100 µm].

The blue arrow represents the healthier neurons while the black arrow represents GFAP staining [Scale bar 100 µm].

GFAP: Glial fibrillary acidic protein

## Discussion

Neurological disorders are some of the important causes of concern that need to be addressed on priority. Scientists are continuously trying to screen compounds for their better efficacy, safety, and cost-effectiveness. Nowadays more attention is being paid to natural products and novel anti-oxidants, as therapeutics for addressing neurological ailments (26 - 28). We, thinking on the same line, investigate neuro neuro-protective potential of PIN against ISO-caused neurotoxicity in Swiss albino mice. We found that PIN significantly attenuates ISO-induced oxidative stress, neuro-inflammation, neurodegeneration, and cognitive impairment in Swiss albino mice.

ISO causes activation of NOX which is accountable for the uncontrolled production of ROS (29). Excessive amount of ROS causes activation of NF-κB and is associated with neuro-inflammation and oxidative damage, which is the main reason for the decrease in neural capacity and cognitive functions (3). In order to confirm cognitive dysfunction, we performed three different behavioral parameters like, FST, EPM, and rotarod. In FST, we found decreased swimming and climbing time and increased immobility time in the ISO-treated group, which showed depressive behavior (30). Contrary to that when we treated with PIN (50 and 100 mg/kg) augmented swimming and climbing time and reduced depressive behavior were observed. To confirm anxiety levels we performed the EPM test (31) and found that ISO-treated mice spent more time in the closed arm and showed more entries to the closed arm. On treatment with different doses of PIN, we observed a notable increase in the time spent and entries in the open arm. This showed the anti-anxiety effect of PIN. The rotarod test was also performed to evaluate motor coordination and early detection of cerebellar dysfunction (32, 33). In this test, ISO-treated mice showed a notable reduction in time on permanence, whereas PIN-treated groups showed a notable increase in the time on permanence. The results of these behavioral parameters are in accordance with the previously reported studies (30, 31, and 33). ISO administration causes oxidative stress by activation of NADPH oxidase (29). Activation of NADPH oxidase causes neurotoxicity by excessive production of ROS (34). ROS are thought to be involved in secondary neuronal damage as they cause protein damage, DNA damage, and membrane lipids peroxidation. Under normal physiological conditions, a balance is maintained between ROS production and anti-oxidants like SOD, catalase, and reduced glutathione, which eliminates the excess amount of ROS (34). In our study, ISO (15 mg/kg) administration decreased the levels of CAT, GSH, and SOD and increased the MDA level in the hippocampus. 50 mg/kg and 100 mg/kg doses of PIN significantly increased these anti-oxidant enzymes and decreased the MDA level, thus maintaining the anti-oxidant level to normal. Our findings are in line with an earlier study which demonstrated that administration of walnut protein lysates produces neuroprotection via maintaining the normal concentration of anti-oxidant enzymes in the brain (35). 

It is proven that neuroinflammation has a key role in the pathophysiology of many neuropathies like Alzheimer’s disease, seizure, Parkinsonism, etc. (36). It is well understood that NF-κB has an essential role in inflammation and degeneration of neurons (37). Under normal pathophysiological homeostasis, NF-κB is found in cytoplasm in its dormant form. In response to oxidative damage, NF-κB moves into the nucleus and causes transcription of the pro-inflammatory cytokines (38). In our study, we also found that ISO elevated the NF-κB expression in the brain (29) along with increased TNF-α and IL-6 levels. However, IL-10 showed a reverse pattern (Figures 5 and 7). Treatments with PIN (50 and 100 mg/kg) down-regulated the NF-κB expression (in the hippocampus and cortex) in addition to elevating the levels of TNF-α and IL-6 and reducing the level of IL-10 in the hippocampus. The *per se *group did not show any change in NF-κB and cytokine levels (Figures 7 and 5, respectively).

The brain mainly comprises neurons and neuroglial cells (39). In the healthy condition, glial cells remain in the inactive form and are in contact with neurons (40). Under stressed conditions, glial cells produce inflammatory cytokines, as seen in Alzheimer’s disease (41). *In silico *analysis is an advanced technique for assessing the substrate’s binding affinity with the protein at the molecular level. PIN interacted well with GFAP. This prompted us to investigate the *in vivo* potential of PIN in reducing GFAP expression in comparison to ISO. In order to confirm the expression of microglia, we performed IHC of GFAP (a microglia marker). We found that ISO administration caused GFAP overexpression in the hippocampus and cortex. Treatment with PIN reduced GFAP overexpression in the hippocampus and cortex as shown in Figure 8. Based on our findings, we thus can say that PIN is quite effective in the treatment of neuroinflammation by reversing the ISO-induced astrocytosis (GFAP, an activated astrocytes marker) and overexpression of NF-κB along with the neuroinflammatory cytokines in Swiss albino mice brains.

Alteration in the normal concentration of neurotransmitters in the brain causes behavioral changes, cognitive dysfunction, and neurodegeneration (42). AchE, a cholinergic enzyme, manages the concentration of acetylcholine (43). ISO causes reversible inhibition of AchE (7). AchE inhibition can cause alteration in cholinergic transmission. In this study, ISO administration reduced the AchE activity. Reduction in AchE activity caused an elevation in the level of acetylcholine leading to neurological disorders (44). On the contrary, treatment with PIN (50, 100 mg/kg) increases the activity of AchE, as shown in Figure 5D. Our findings are well supported by the earlier findings (3). Apart from AchE activity, BDNF is an important neurotrophin that is required for neuronal and glial development, neurotransmission, and neuroplasticity (45, 46). Deficiency of BDNF level is responsible for neurological disorders, like Alzheimer’s disease, vascular dementia, brain trauma, and stroke (47, 48). In our study, treatment with ISO caused a decrease in the level of BDNF; which on treatment with PIN (50 and 100 mg/kg) gets reversed to normal, as shown in Figure 5E. 

After the insight of oxidative stress, inflammation, modification of neurotransmitters, and cognitive changes, we also looked for histological changes in the hippocampus and cortex. We found that ISO induced significant alteration in the neuronal structure of the hippocampus and cortex and caused altered neuronal architecture, pyknosis, vacuolization, and neuro-degeneration (Figure 6) (3, 29, 49). Treatment with PIN notably reversed these histopathological alterations and thus worked as a potential neuroprotective agent.

Furthermore, it is well-recognized that elevated ROS and low levels of anti-oxidants cause neuroinflammatory and cognitive impairment (50, 51). ROS elevate the levels of MDA and inflammatory cytokines and reduce the anti-oxidants, which activates NF–κB, which is involved in the control of different gene transcription responsible for amelioration of memory diseases such as Alzheimer’s (51). Overexpression of NF–κB by active form of IKK2 results in depletion of BDNF levels. Decreased BDNF level is involved in neurodegeneration and memory impairment (5). Zhuang *et al*. reported that IL-33 inhibited the BDNF expression through NF–κB (52). A previous study reported that oxidative stress induced neuroinflammation by NF-κB activation and GFAP overexpression (53). In the current study, ISO administration notably increased the NF–κB and GFAP expression and decreased the BDNF level. Treatment with PIN and propranolol reduced the level of NF–κB and GFAP expression and increased the level of BDNF. As a result, we can conclude that D-Pinitol reduces inflammatory reactions and improves cognitive dysfunction via modulation of NF- κB/BDNF/GFAP signaling pathways in order to protect against neurotoxicity.

## Conclusion

Based on our findings, we can conclude that isoproterenol caused neurological toxicity in terms of oxidative damage, inflammation, and histological changes via alteration in NF-κB, GFAP, AchE, BDNF, anti-oxidant activities, inflammatory markers, and histology of the brain. PIN 100 mg/kg notably reversed these altered parameters towards the normal. The results of behavioral studies, like the forced swim test, elevated plus maze test and rotarod test substantially strengthened these biochemical and histopathological findings. Our results of the *per se* group also suggest that PIN alone did not produce any adverse effects. In conclusion, our findings on PIN clearly established its neuroprotective role in ISO-induced neurodegeneration in Swiss albino mice.

## Authors’ Contributions

A K performed the experiment and compiled and interpreted the data; S S assisted in creating illustrations, figures, and their interpretation; A D helped with software and calculations, M MA helped in drafting the manuscript; M AS reviewed and edited the manuscript; S EH conceptualized and supervised the whole project.

## Funding Source

This research did not receive any specific grant from funding agencies in the public, commercial, or not-for-profit sectors.

## Conflicts of Interest

There are no conflicts of interest.
